# Detection rate of SARS-CoV-2 RNA in relation to isolation time and environmental surface type

**DOI:** 10.3389/fpubh.2022.957827

**Published:** 2022-09-14

**Authors:** Weijie Sun, Huimin Chen, Shuan Tao, Na Li, Yao Xu, Yewei Fang, Luyan Chen, Wei Liang, Gang Cao

**Affiliations:** ^1^Department of Clinical Laboratory, Ningbo First Hospital, Ningbo, China; ^2^School of Medicine, Medical School of Jiangsu University, Zhenjiang, China; ^3^Laboratory Medical School, Bengbu Medical College, Bengbu, China; ^4^School of Medicine, Medical School of Ningbo University, Ningbo, China; ^5^Department of Pain Clinic, Ningbo First Hospital, Ningbo, China

**Keywords:** SARS-CoV-2, COVID-19, the detection rate, environmental surface, material

## Abstract

Severe acute respiratory syndrome coronavirus (SARS-CoV-2) causes environmental contamination *via* respiratory droplets and persists on contaminants and environmental surfaces for anywhere from a few hours to 6 days. Therefore, it is particularly important to understand the transmission and containment of SARS-CoV-2 on the surface of objects within isolated environments. In this study, 356 environmental surface samples were collected and 79 tested positive, with the highest contamination rate (56.96%) in the wood category (bedside tables, wood floors, and walls). This study revealed differences in the detection rates of environmental surfaces in hospitalized and discharged rooms of patients with confirmed COVID-19 in 2 isolated settings (A: *p* = 0.001; B: *p* = 0.505) and suggested that environmental contamination may be an important route of virus transmission, providing a reference to guide the enhancement of ventilation, the use of hotel isolation model, the advocacy of cotton masks, and the effective suppression of virus transmission.

## Introduction

Severe acute respiratory syndrome coronavirus-2 (SARS-CoV-2) has spread rapidly globally, and as of 5 May 2022, more than 510 million confirmed cases of coronavirus disease (COVID-19) and over 6.25 million people have reportedly died worldwide (1). According to the current data, the transmission of SARS-CoV-2 is mainly about (2–4) occurring through infectious respiratory droplets and aerosol-producing medical procedures, direct contact with the secretions of infected persons, or indirect contact with contaminated surfaces. Airborne transmission of SARS-CoV-2 is the main route of transmission: droplets and aerosols. Virus particles encased in mucus balls are released into the air when a patient with COVID-19 opens his mouth to speak, sneezes, coughs, or breathes (5). According to the World Health Organization (WHO) and the Centers for Disease Control and Prevention (CDC), the particles are judged as droplets when they are larger than 5 μm in diameter and as aerosols when they are smaller than 5 μm in diameter (6, 7). Approximately 20% of patients are reported to produce 80% of the aerosols in the room, while approximately 10–20% of COVID-19 causes about 80–90% of infections (8, 9), but little is known about the potential for transmission through contact with surfaces or objects contaminated with airborne SARS-CoV-2 (10).

Symptomatic and asymptomatic patients or infected animals can detect viral (11) on various surfaces of contaminated environments, and therefore, contact with contaminated surfaces may also play an important role in infecting SARS-CoV-2. Due to the strong infectivity and high concealment of the virus, the environmental surface material in the hospital ward and in the shelter hospital ward may be severely contaminated with (12) by SARS-CoV-2. Environmental surface contamination can trigger contact transmission, leading to the occurrence of COVID-19 and the spread of (13). The (14) human coronavirus has been reported to remain infectious in aerosols for 3 to 16 h and survive 65% surfaces at room temperature and relative humidity, and previous studies described (15) retention of SARS-CoV-2 on surfaces ranging from several hours to 6 days.

In this case, this study detected SARS-CoV-2 on the surfaces of objects in isolation rooms, explored the impact of isolation environment, contamination time, and exposure to surface material on SARS-CoV-2 virus detection rate, and understood the role of environmental surface materials contaminated with SARS-CoV-2 in disease transmission, and the factors that cause SARS-CoV-2 persistence on the surface will help to more accurately estimate the risk of exposure transmission and inform the improvement of isolation environments.

## Materials and methods

### SARS-CoV-2 surface sampling

The study enrolled 27 inpatients and discharged 29 patients between 19 April 2022 and 26 April 2022 at two isolation sites, Dapeng Mountain Isolation Ward (A) and Shelter Hospital (B), Ningbo, Zhejiang Province.

Environmental contaminants such as bedside tables, pillows, switches, and cell phones were sampled in rooms of patients with SARS-CoV-2 confirmed by real-time fluorescent quantitative PCR (RT-PCR), disinfected with 75% alcohol before and after, and repeatedly rinsed and wiped. Room surfaces of ward floors, door handles, guardrails, and wall panels were sampled, disinfected before and after spraying with 2,000 mg/L chlorine-containing disinfectant, and then repeatedly rinsed and wiped with water.

### Quantitative reverse transcription by polymerase chain reaction

Viral RNA extraction and qRT-PCR for SARS-CoV-2 were performed as prescribed by the piece of research (16). RNA was extracted using MagPureViral DNA/RNA Kit (Jiaxing; Zhejiang; China), and the N gene and open reading frame (ORF 1ab) regions of SARS-CoV-2 were amplified by real-time quantitative PCR according to the 2019-nCoV Nucleic Acid Detection Kit (Mingde Biotechnology Co., Ltd; Wuhan, China) standards (Cycle threshold Ct < 40 as positive for SARS-CoV-2 RNA and Ct > 40 as negative). We classified Ct < 35 as high viral load and Ct > 35 as low viral load in this study. A positive double-target gene or a positive single target gene was determined as SARS-CoV-2 virus detection, while a negative double-target gene was determined as non-detection.

### Statistical analysis

Environmental samples of each type from the same patient are grouped together, and a COVID-19 patient is considered a positive sample patient when the environmental sample examined by that patient is contaminated. For each contaminant type, the positivity rate was calculated as (number of positive specimen patients)/(total number of patients) unless otherwise stated. Differences in specimen detection rates were compared using the chi-square test or Fisher's exact test, and *P* < 0.05 was considered statistically significant. All statistical analyses were performed using SPSS version 22.0 (SPSS), and statistical graphs were drawn using GraphPad Prism 8.0 (GraphPad Software, Inc.).

## Result

### SARS-CoV-2 RNA was detected in environmental samples collected from hospitalized and discharged COVID-19 patients

A total of 180 environmental samples were collected from 27 hospitalized patients and 176 from 29 discharged COVID-19 patients, and the sampling details and SARS-CoV-2 RNA test results are shown in Table 1.

**Table 1:** Results of different environmental samples from 56 COVID-19 patients.

	**Hospital ward**		**Shelter**	
**Item**	**Hospitalization**	**Discharge from hospital**	**Hospitalization**	**Discharge from hospital**
Door handle	0/15	3/20	—	—
Bedside table	6/15	14/20	1/12	2/9
Pillow	1/15	5/20	3/12	1/9
Wooden floor	8/15	14/20	—	—
Bidet toilet	1/15	2/20	—	—
Patient mobile	0/15	—	1/12	—
Wall	0/15	0/20	—	—
Switch	0/15	5/20	6/12	3/9
Guardrail	—	—	2/12	1/9

COVID-19 patients are divided into two groups: hospitalization and discharge.

The data in the table are positive samples accounting for the number of patients tested, in which “–” indicates that the item did not participate in environmental sampling.

**Figure 1:**
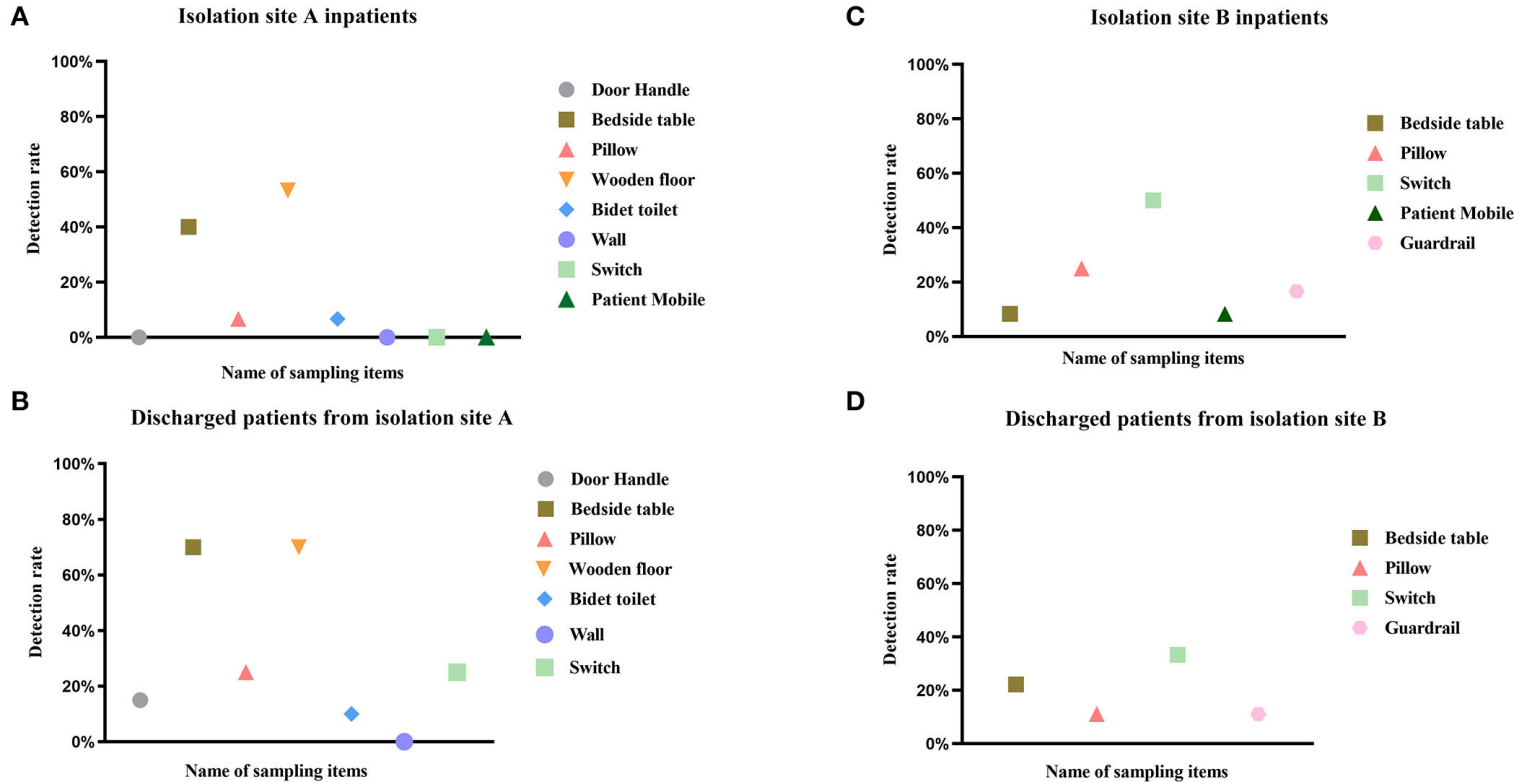
Detection rate of SARS-CoV-2 RNA in different environmental samples. This figure depicts the detection rate of SARS-CoV-2 RNA in environmental samples of COVID-19 patients hospitalized **(A,C)** and discharged **(B,D)** from isolation site A and isolation site B. The positivity rate was calculated as (number of patients with positive specimens)/(total number of patients), and detailed data are shown in Table 1. Different colors represent different sampled items, and the same color is the same category.

1. Wooden floor samples had the highest positivity rate in the inpatient (53.3%) and discharge (70%) groups at isolation site A, followed by bedside table samples (inpatient group: 40%; discharge group: 70%). switch samples had the highest positivity rate in the inpatient (53.3%) and discharge (70%) groups at isolation site B, followed by pillow (25%) and bedside table (22.22%) samples, respectively.

2. Fifty-nine (22.69%) of 260 samples from isolation site A were positive, while twenty (20.83%) of 96 samples from isolation site B were positive. There was no difference in the potential for transmission of the virus to the surrounding environment between patients in isolation sites A and B (*p* = 0.413), although there were differences in their environments. There was a temporal difference in environmental surface sampling between inpatients and discharged patients, and by statistical analysis of patients at the two isolation sites, It was found that there was a significant difference in virus detection rates between the two environmental samples for patients at isolation site A (*p* = 0.001) and no difference in environmental surface virus detection rates for patients at isolation site B (*p* = 0.505) (Figure 1).

### Assessing SARS-CoV-2 contamination rates on surfaces of different material types

**Figure 2:**
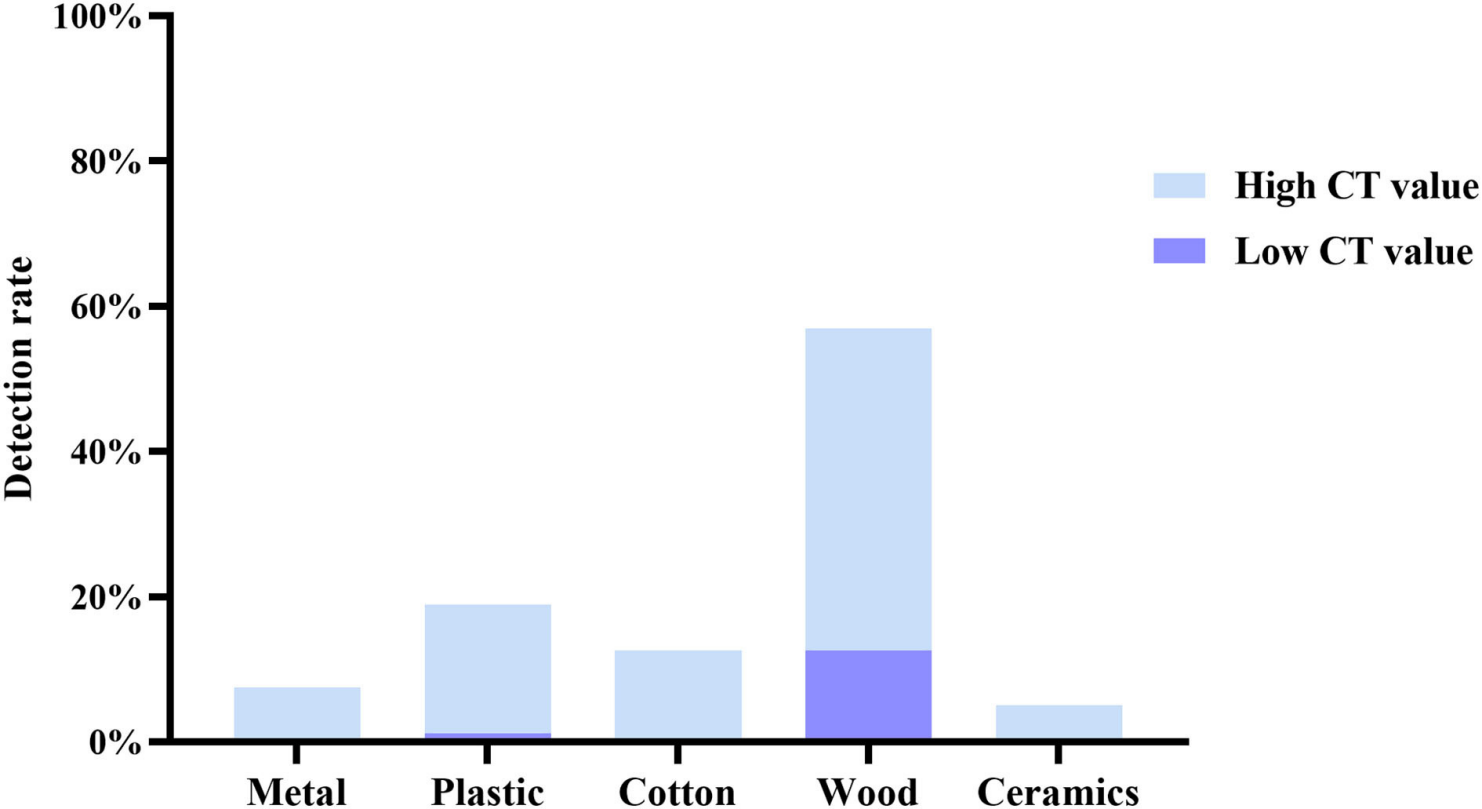
Detection rate of SARS-CoV-2 RNA in environmental specimens of various materials.

We classified all test samples with reference to the review (17), including metal (door handle and guardrail), plastic (switch and patient phone), cotton (pillow), wood (bedside table, wood floor, and wall), and ceramics (bidet toilet). According to the SARS-CoV-2 detection gene cycle threshold, it was further divided into high Ct value(Ct > 35) group and low Ct value(Ct < 35)group. The total number of positive samples in this test was 79 cases, including 11 cases in the high Ct value group and 68 cases in the low Ct value group. The detection rate of wood RNA viruses was the highest (44.3% with high Ct value and 12.66% with low Ct value), followed by the pollution of plastics, which is relatively high (17.72% with high Ct value and 1.27% with low Ct value) (Figure 2).

## Discussion

Despite unprecedented world efforts to control the spread of COVID-19 and its cause, SARS-CoV-2, the number of confirmed cases has sadly continued to increase over the past 3 years and the mutation of SARS-CoV-2 could not have been anticipated. In the current SARS-CoV-2 pandemic, contact with surfaces contaminated with the virus is considered a non-negligible route of transmission (18). Due to the potential for contamination events in a range of materials (19), this would increase the concern of patients and staff about the potential for transmission through contaminants. In addition, the survival time of a virus in an environment other than its host is equally critical in allowing its transmission (17). Next, we monitored 356 environmental samples collected from 56 COVID-19 patients in an isolation ward setting in a hospital and a shelter hospital, and SARS-CoV-2 RNA was detected in 79 (22.19%). Contaminated surfaces included bedside tables, wooden floors, pillows, switches, guardrails, door handles, bidet toilets, and mobile phones.

A higher proportion of contaminated surfaces (22.69%) was detected in mechanically ventilated isolation A wards, although this trend did not reach statistical significance (*p* = 0.413). Studies have shown that SARS-CoV-2 viral load peaks in the first week in patients with COVID-19 (20), with a higher presence of environmental surface contamination. We observed a higher detection rate in inpatients relative to discharged patients in isolation ward A (*P* = 0.001), whereas no statistical significance was shown in the detection rate of environmental sampling specimens in isolation ward B. This was strongly related to the environment in which the patients were treated, with isolation ward A being physically blocked from virus transmission by hotel management, and isolation ward B showing a group living status, combined with poor mechanical ventilation, and no major difference in environmental surface contamination rates between discharged and inpatients. The respiratory-transmitted viral load of inpatients was significantly higher than that of discharged patients, so we advocate more the hotel isolation ward model to block the transmission of SARS-CoV-2 virus, coupled with effective disinfection measures to control COVID-19.

Paton et al. (18) found that SARS-CoV-2 RNA showed high stability on the surface of dry objects, with only a 1 log reduction in recovery over 3 weeks, which could explain the highest detection rate of wood samples in isolation wards.

Previous data (21) showed that SARS-CoV-1 virus can survive on wooden boards for 4 days in a 21°C−25°C environment, with complete decay starting on day 5. Furthermore, since SARS-CoV-2 RNA can survive in aerosols for as long as 3 h (22), the aerosols continuously exhaled by patients end up depositing on wooden floors (8) and bedside tables are frequently touched by patients, which seems to explain the highest detection rate of wood-based samples in isolation wards.

Due to the hydrophilic and woven nature of cotton, virus particles remain in cotton fibers after contamination but are unable to survive on surfaces (18, 23), but SARS-CoV-2 can survive on plastic and metal surfaces for up to 72 h (24), posing some risk of transmission. Increasingly, data (25) found that cotton has the lowest environmental stability to SARS-CoV-2, with a 99.995% reduction in 1h virus titer, and performs more stably on smooth surfaces, such as plastics and metals, persisting in contaminants for 4–5 days (17). We speculate that the half-life of the virus may be strongly correlated with the temperature and humidity of the object surface, but there is no complete statement to explain this phenomenon. In addition, it is based on the fact that viral viability decreases rapidly after exposure to cotton and that the highest positive rate is found on wooden floors, increasing public support for cotton masks and strengthening daily disinfection of different object surfaces in COVID-19 isolation wards, especially wooden floor.

Based on previous statistical analyses (26, 27), excluding the correlation between viral load of clinical samples and viral load of environmental samples, we had a relatively higher incidence of contamination on wood-like and plastic specimen surfaces during illness and more viral load than other environmental samples. The data from this study indirectly show the ability of material types to survive SARS-CoV-2 on surfaces and the potential for some transmission with the possible presence of live virus in the surface of objects. Although the use of RT-PCR to interpret surface sampling results to determine the likelihood of the presence of live virus on surfaces has implications (18), further validation is also needed for positive surface sampling results vs. the presence of infectivity. Thus, although we detected SARS-CoV-2 RNA on the surface, it does not indicate the presence of live virus and whether it is infectious to patients and staff, as infectivity is not related to Ct values (27). In addition, because SARS-CoV-2 detection on the surface of objects does not correlate with their infectivity and activity, the number of respondents tested was limited, for example, in isolation ward B. This also contributes to the slight uncertainty and limitations of this study.

With regard to the prevention and control of SARS-CoV-2 transmission, the CDC in each country provides cleaning and disinfection guidelines with specific strategies, and the results of our study support the CDC's disinfection recommendations. Currently, there is no effective and safe vaccine or antiviral drug for SARS-CoV-2 transmission or for the control of COVID-19, so controlling the source of infection and disrupting the transmission route are key to limiting the spread of the virus. In addition, further research is still needed on how to disinfect and mitigate contamination of environmental surface materials to reduce the risk of exposure.

## Data availability statement

The original contributions presented in the study are included in the article/supplementary material, further inquiries can be directed to the corresponding author/s.

## Author contributions

WS and HC: wrote the original draft, formal analysis, and conceptualization. WL: conceptualization, reviewed and edited the manuscript, and supervision. ST and NL: validation, investigation, and data curation. All authors contributed to the article and approved the submitted version.

## Funding

This study was supported by grants from the Natural Science Foundation of Jiangsu Province (BK20191210), the fifth phase of the 333 Project Scientific Research Project in Jiangsu Province (BRA2019248), and the Jiangsu Commission of Health (H2018073).

## Conflict of interest

The authors declare that the research was conducted in the absence of any commercial or financial relationships that could be construed as a potential conflict of interest.

## Publisher's note

All claims expressed in this article are solely those of the authors and do not necessarily represent those of their affiliated organizations, or those of the publisher, the editors and the reviewers. Any product that may be evaluated in this article, or claim that may be made by its manufacturer, is not guaranteed or endorsed by the publisher.
